# Rapid exacerbation featuring acute leukemoid reaction after retrolaparoscopic nephrectomy: a rare case report of renal cell carcinoma with postoperative comprehensive genomic profiling

**DOI:** 10.1186/s12957-020-01926-4

**Published:** 2020-07-06

**Authors:** Xuhui Zhang, Lijuan Yan, Xiaobin Yuan, Tao Bai, Lei Zhang, Shuaihong Han

**Affiliations:** 1 Department of Urology, First Hospital of Shanxi Medical University, No. 85, Jiefangnan Road, Taiyuan, 030001 Shanxi China; 2 Shanxi Cancer Institute, Shanxi Cancer Hospital, Taiyuan, 030000 Shanxi China; 3 Department of Pathology, First Hospital of Shanxi Medical University, Taiyuan, 030001 Shanxi China

**Keywords:** Renal cell carcinoma, Acute leukemoid reaction, Comprehensive genomic profiling, Gene mutation, Granulocyte colony-stimulating factor

## Abstract

**Background:**

Rapid lethal exacerbation and recurrence featuring acute leukemoid reaction (ALR) after retrolaparoscopic radical nephrectomy (RN) is a relatively rare clinical incident. Performing the reoperation for the patient and analyzing the tissue-based genetic mutation information postoperatively are a skill-demanding and meaningful task, which have been even more rarely reported.

**Case presentation:**

We present a case with a large right renal mass (13.0 × 10.0 × 8.0 cm). This 71-year-old male patient underwent the retrolaparoscopic RN in our department. The operation was technically precise and successful with final pathological diagnosis of hybrid (clear cell and papillary type) renal cell carcinoma (RCC). However, 10 days after the patient was discharged, he was readmitted with the chief complaint of high fever with severe right flank pain. CT scanning revealed that right retroperitoneal hematoma and the blood routine showed the dramatic elevation of white blood cell count (WBC). Even though the immediate broad-spectrum antibiotics were administered without delay and subsequent percutaneous puncturing and drainage was performed, the patient’s condition still exacerbated rapidly. In spite of the reoperation of hematoma evacuation, the patient died of multiple organ failure 10 days after the reoperation. The pathological result of reoperation showed the necrotic and hematoma tissue blended with RCC tumor cells (nuclear grading III), and both of the postoperative tissue-originated comprehensive genomic profiling by using the specimens from the RN and reoperation respectively indicated significant mutations of some oncogenes which might have potential relevance with ALR. Besides, both of the immunohistochemical (IHC) staining results from primary surgical renal mass and reoperative resected tissue revealed the positive expressions of granulocyte colony-stimulating factor (G-CSF).

**Conclusions:**

ALR may be a predictor of poor prognosis in patients with RCC, and comprehensive genomic profiling as well as the alterative expression of G-CSF can help to provide potential valuable genetic etiological information and evidence for guiding the potential effective molecular-targeting therapy.

## Background

As the third most common malignancy in the genitourinary system, renal cell carcinoma (RCC) affects about nearly 3% of all adult malignancies [1] and led to 14,400 tumor-related death events and 63,990 new diagnosed cases in the USA last year [2]. For the last three decades, the survival improvement of RCC mainly attributed to the timely implementation of surgical interventions, especially the laparoscopic surgery [3, 4]. Acute leukemoid reaction (ALR) is defined as reactive leukocytosis exceeding 40 × 10^9^/L, with a significant increase in early neutrophil precursors, and can be a paraneoplastic manifestation of various malignant tumors [5]. ALR has been reported in tumors of the stomach, colon, liver, gall bladder, pancreas, lung, bladder, bone, and thyroid gland [6–11]. There are a few published papers that described ALR in patients with renal mass [12–14], but the authors only focused on the clinical findings with the absence of subsequent genetic mutation analysis. Here, we present a rare case of a large renal tumor (13.0 × 10.0 × 8.0 cm) with postoperative ALR and rapid lethal deterioration featuring the tissue-originated comprehensive genomic profiling. To our knowledge, our case is the first report that combined the perioperative clinical description with the genetic investigation by far.

## Case presentation

A 71-year-old male patient was admitted into our hospital with chronic right flank pain. The abnormal computed tomography (CT) image presentation of right renal mass, revealed by occasional health examination, accompanied with the complaint of intermittent nausea. Besides, his past medical history also included diabetes mellitus (type 2) for 10 years and significant hypertension for 12 years, ranging from 140–160/90–95 mmHg, fluctuated periodically. Regular combined subcutaneous injection of short-acting insulin and insulin glargine was recorded for the treatment of diabetes mellitus, and the patient claimed that the serum glucose level was controlled satisfactorily all the time. As for hypertension, nifedipine sustained-release tablets were prescribed as the oral therapy by the physician, but the patient admitted that he failed to follow the long-term regular medication.

The patient’s height was 171.0 cm, and the body weight was 65.0 kg. The BMI was 22.23 kg/m^2^. The laboratory tests presented the elevated level of white blood cell (WBC) count (29.9 × 10^9^/L). The red blood cell (RBC) count and other vital parameters were all within the reference range.

**Fig. 1:**
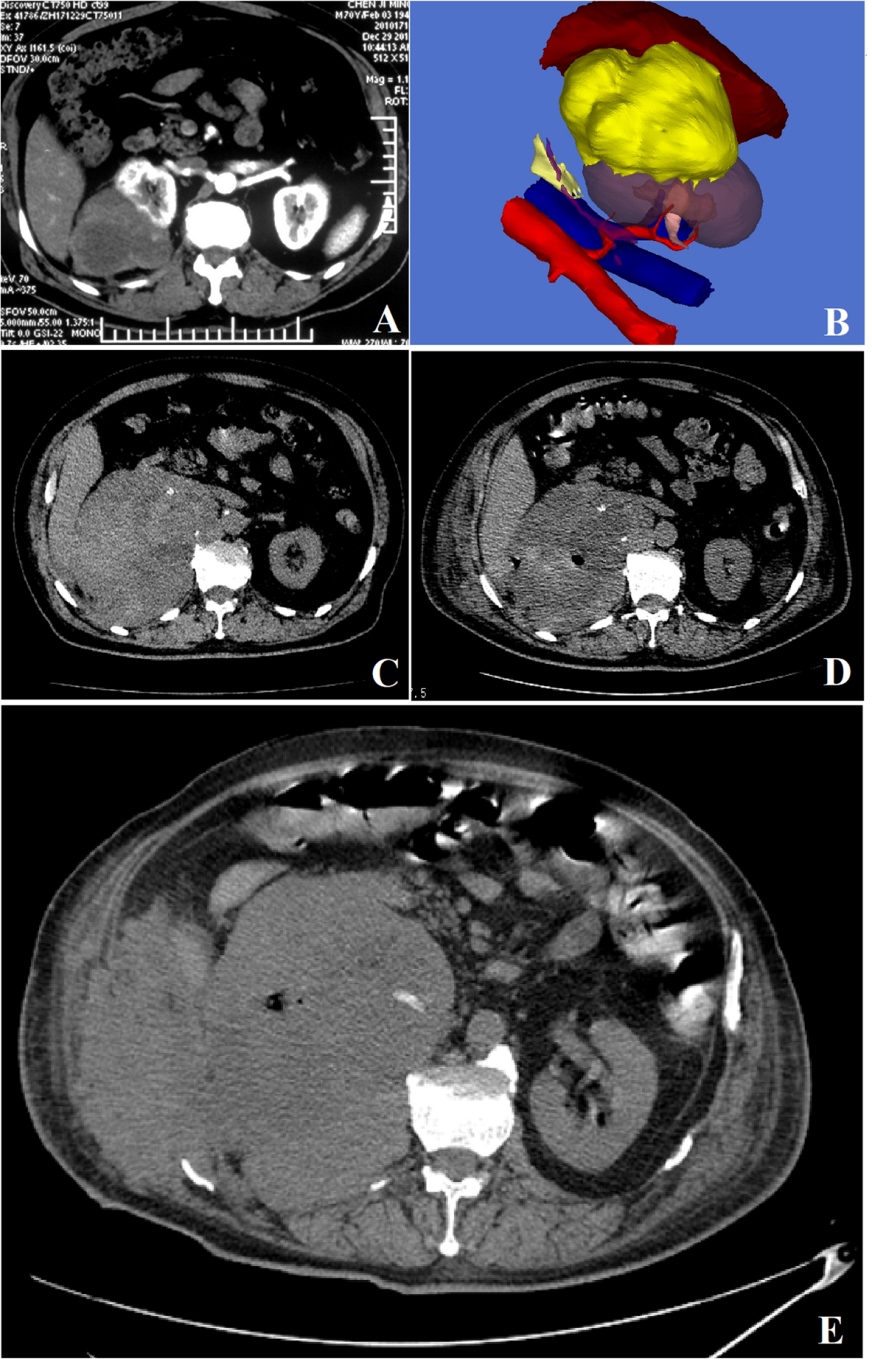
**a** The primary CT findings when admission showed the right renal mass with the size of 13.0 × 10.0 × 8.0 cm. **b** The reconstructed three-dimensional digital model (3D-DM) marked by using different colors (yellow color: the renal mass). **c** The rechecked CT findings when readmission showed a right retroperitoneal hematoma with the size of 16.3 × 13.2 × 10.4 cm. **d** The rechecked CT scanning result after the percutaneous puncturing and drainage showed no significant shrinkage for the retroperitoneal hematoma. **e** The day before his clinical death, the rechecked CT scanning images showed that the rapid retroperitoneal cavity metastasis with obvious sign of puncturing track implantation

As for the medical image examinations, the routine chest X-ray image did not reveal the abnormal findings. The strong and heterogeneous enhancement pattern from the dual-source 64-slice enhanced CT-scanned films including arterial phase, venous phase, and excretory phase indicated the radiologic diagnosis of right malignant renal tumor with the size of 13.0 × 10.0 × 8.0 cm (Fig. 1a) [15].

In order to facilitate the surgical orientation and improve the manipulating accuracy, the data from CT images was extracted, and the three-dimensional digital model (3D-DM) was reconstructed. The copied information was analyzed and reconstructed into the 3D-DM by using a postprocessing software named three-dimensional medical image reconstructing and guiding system (3D-MIRGS, China). The retroperitoneal space along with critical anatomic structures including renal tumor, the relevant vasculature, the kidney, and the renal collecting system on the affected side were reconstructed and marked by using different colors simultaneously (Fig. 1b).

The retrolaparoscopic RN was performed under the assisted navigation of 3D-DM at the Department of Urology. The right kidney along with the right adrenal gland was dissected and excised completely. The hemostasis was achieved carefully.

**Fig. 2:**
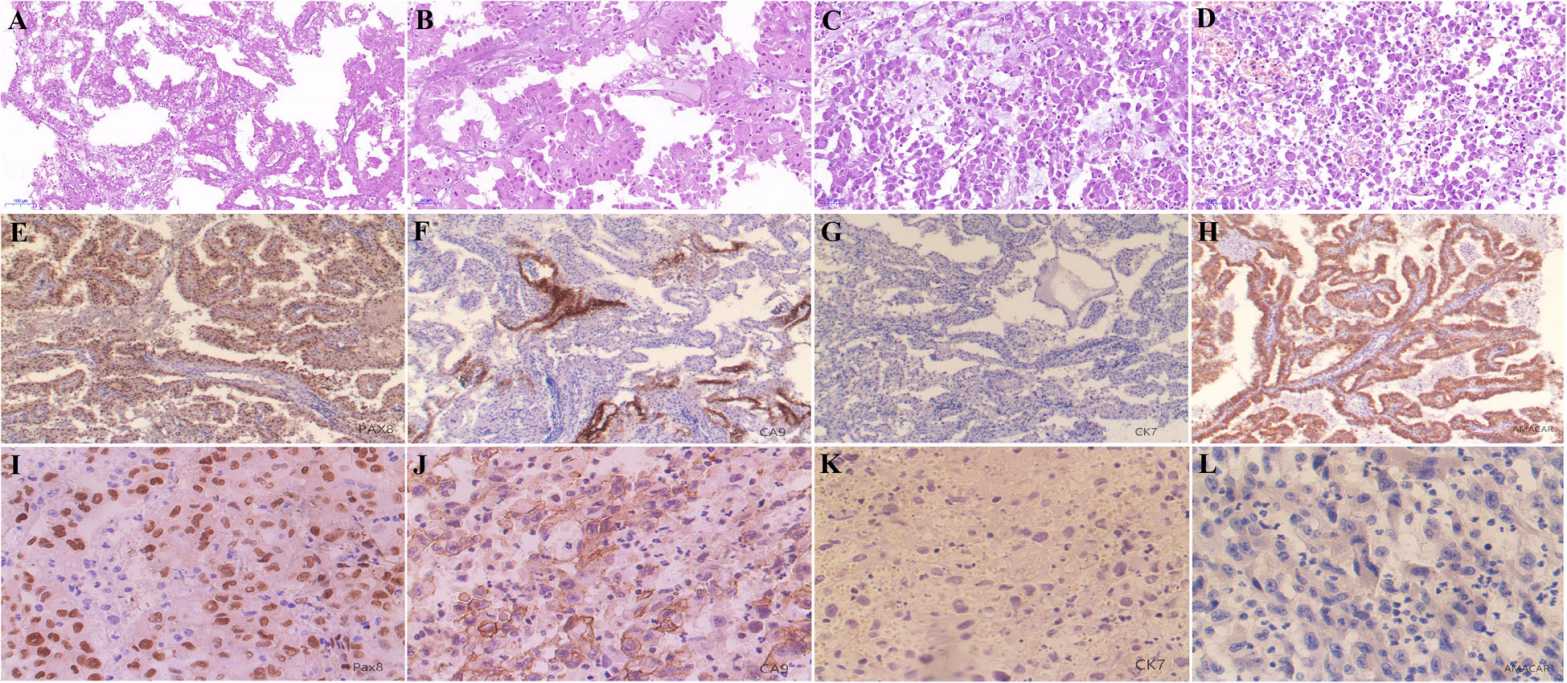
**a**, **b** The pathological diagnosis of first operation was hybrid (clear cell and papillary type) renal cell carcinoma (nuclear grading III). **c**, **d** The final pathological result of reoperation showed the necrotic and hematoma tissue blended with RCC tumor cells (nuclear grading IV). **e**–**h** The results of immunohistochemical staining from the specimens of 1st operation. (**e** Pax8, **f** CA9, **g** CK7, and **h** AMACAR). **i**–**l** The results of immunohistochemical staining from the specimens of 2nd operation. (**i** Pax8, **j** CA9, **k** CK7, and **l** AMACAR)

The operative time was 1 h and 30 min, with no intraoperative complications happened. The estimated blood loss was about 20 mL. The size of resected tumor was 10.0 × 8.0 × 6.0 cm. The final pathological diagnosis was hybrid (clear cell and papillary type) renal cell carcinoma (nuclear grading III) (Fig. 2a, b). WBC count fell back to the level of 10.6 × 10^9^/L at the first day postoperatively, which further dropped down to 7.3 × 10^9^/L 2 days later. The recovery course was uneventful, and the patient was discharged after 7 days postoperatively.

**Fig. 3:**
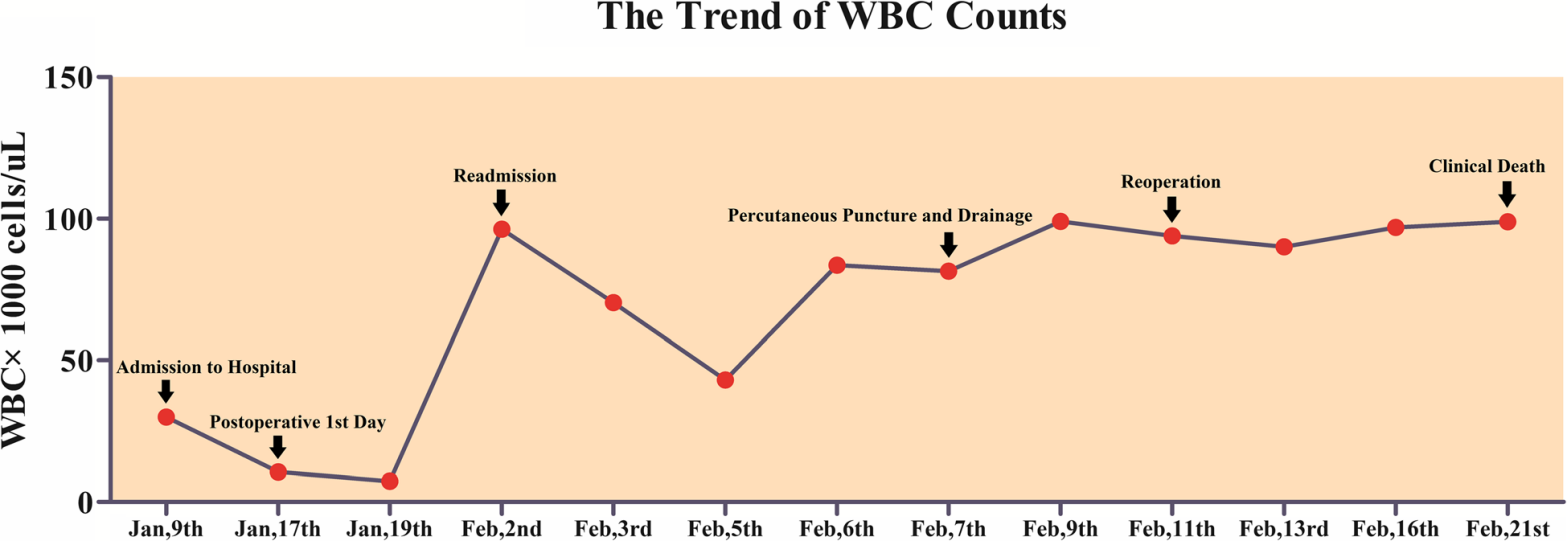
The trend of WBC counts from the patient’s admission to his clinical death

Ten days after the patient was discharged, he was readmitted with the chief complaint of high fever with severe right flank pain. CT scanning revealed that right retroperitoneal hematoma (Fig. 1c) and the blood routine showed the dramatic elevation of WBC count (96.3 × 10^9^/L). At the same time, the body temperature was high (37.9 to 40.5 °C). ALR was established. He was treated with systemic antibiotics and the temperature decreased, but the WBC count still remained on high level. After the consultation of multi-disciplinary team and department discussion, we decided to perform the percutaneous puncturing and drainage for him after a 5-day systemic antibiotic therapy. Even though there was bloody drainage fluid with the amount of about 300 mL each day, the patient’s condition still exacerbated rapidly and the CT scanning result showed no significant shrinkage for the retroperitoneal hematoma (Fig. 1d). Five days after the drainage, the patient’s symptom of right flank swelling and pain still aggravated significantly. In spite of the immediate reoperation of hematoma evacuation, the patient died of multiple organ failure 10 days after the reoperation eventually. The day before his clinical death, the rechecked CT scanning images showed the rapid retroperitoneal cavity metastasis with obvious sign of puncturing track implantation (Fig. 1e). From the admission to clinical death, the trend of WBC counts can be seen in Fig. 3.

The final pathological result of reoperation showed the necrotic and hematoma tissue blended with RCC tumor cells (nuclear grading IV) (Fig. 2c, d). Furthermore, the immunohistochemical staining from the specimens of two operations was performed to evaluate the expressions of 4 markers. Primary antibodies used in the study included paired box 8 (Pax8) (Santa Cruz Biotechnology sc-514352, Dallas, TX, USA), carbonic anhydrase 9 (CA9) (Santa Cruz Biotechnology sc-365900, Dallas, TX, USA), cytokeratin 7 (CK7) (Santa Cruz Biotechnology sc-23876, Dallas, TX, USA), and α-methylacyl-coenzyme A racemase (AMACAR) (Santa Cruz Biotechnology sc-515623, Dallas, TX, USA). UltraView™ DAB detection kit was purchased from Ventana (Arizona, America). All immunohistochemistry assays were performed on the Roche BenchMark XT fully automatic IHC/ISH instrument by optimized protocols. The results of immunohistochemical staining from the specimens of two operations all indicated that the tumor cells were immunopositive for Pax8 and CA9, but negative for CK7 and AMACAR (Fig. 2e–l).

**Fig. 4:**
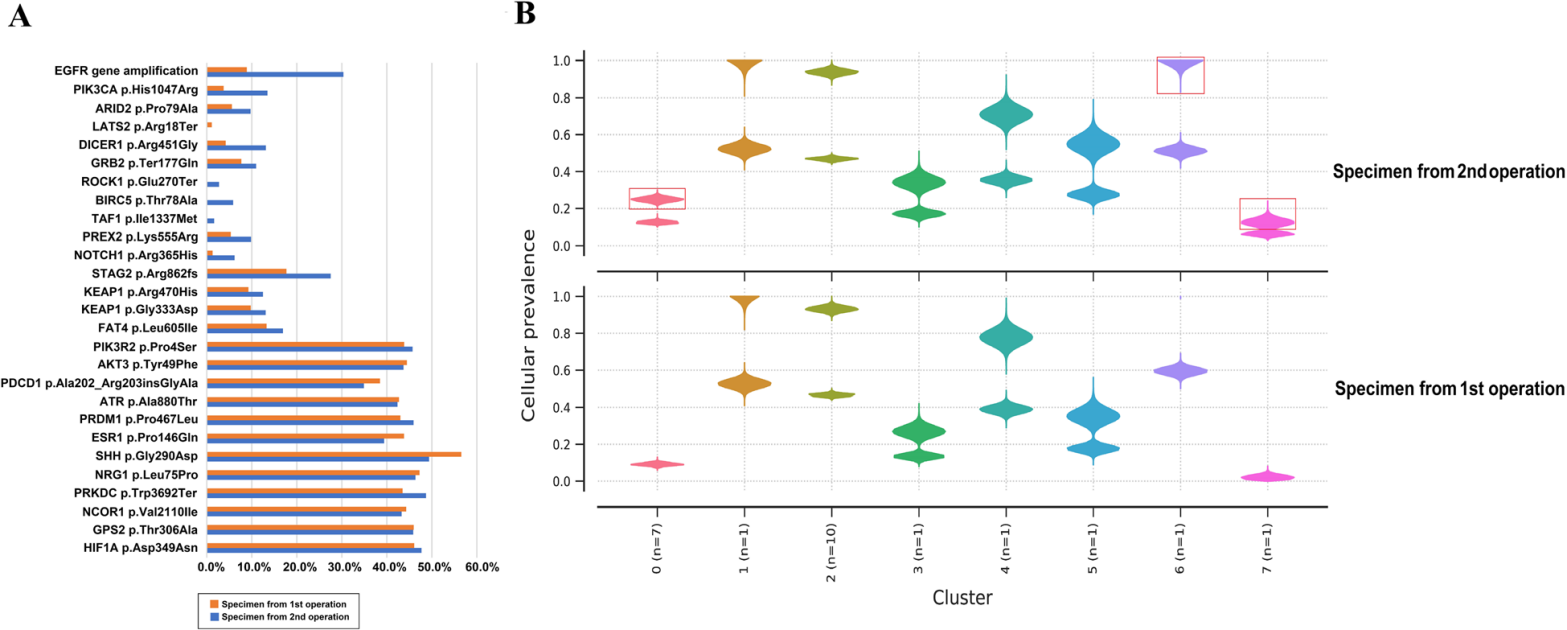
The analysis of postoperative comprehensive genomic profiling. **a** The summary of gene mutation abundance indicated that most of the gene mutation abundance from the specimen of reoperation was higher than the specimen of primary operation. **b** The mutation was divided into 8 clones (0–7) according to the information of mutation abundance and mutation types. Most clones of reoperation specimen were derived from the primary tumor specimen, and some added clones can be seen (marked in red frame), indicating that the metastases evolved from the primary foci

**Fig. 5:**
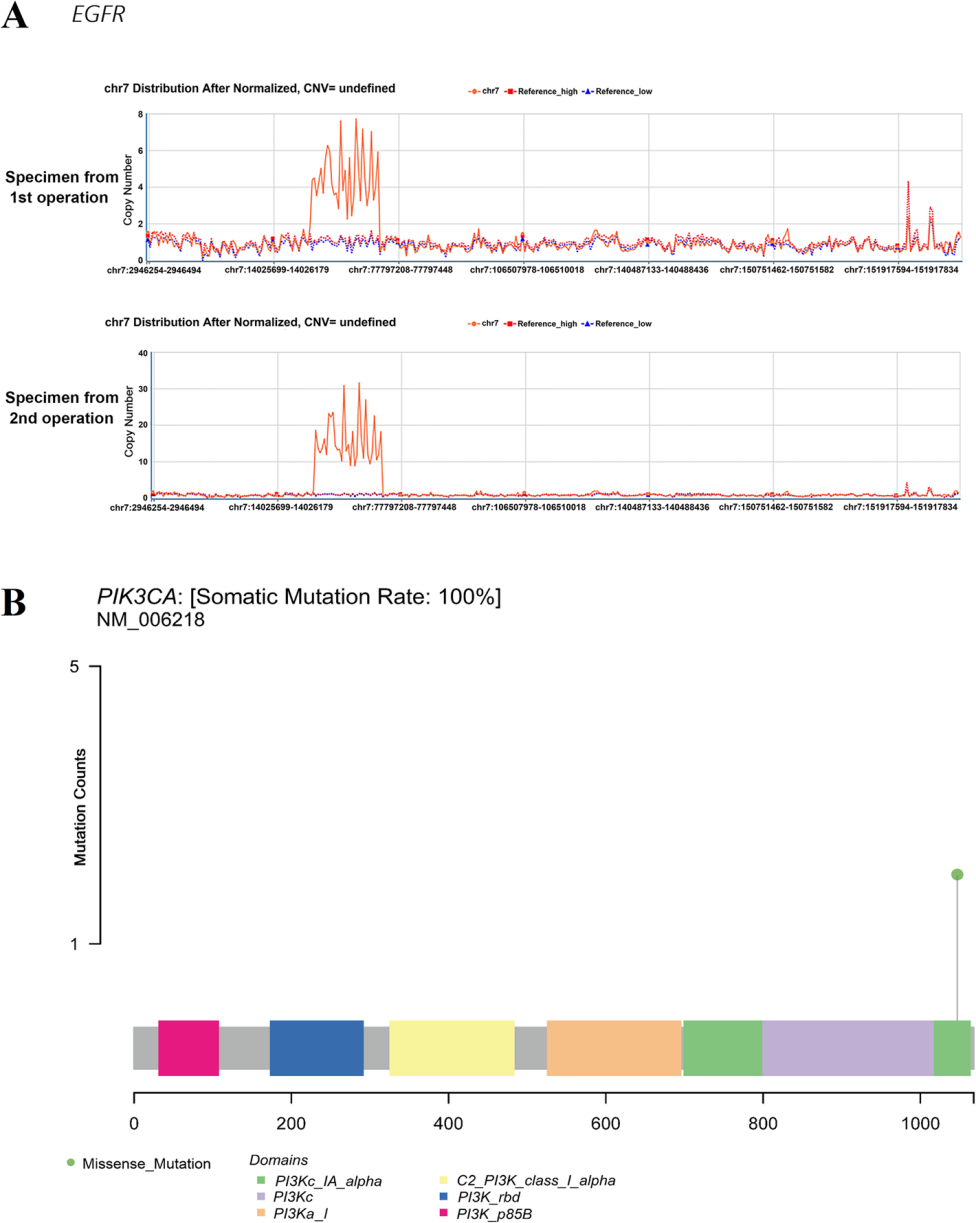
**a** The proliferative mutation of *EGFR*. **b** The missense mutation of *PIK3CA*

After the death of the patient, we retrieved part of the surgical specimens from two operations he underwent and delivered them to a biochemical company (Life Healthcare, China) which provides the service of genetic testing. Both of the postoperative tissue-originated comprehensive genomic profiling by using the specimens from the RN and reoperation respectively indicated significant mutations of several genes (Fig. 4 and the detailed data of comprehensive genomic profiling is shown in Supplemental. 1). There are 7 genes’ mutation abundance that showed a statistical difference between two operations’ specimens, featuring the most dramatic elevated mutation of *phosphatidylinositol-4,5-bisphosphate 3-kinase catalytic subunit alpha (PIK3CA)* and *epidermal growth factor receptor (EGFR)* (Table 1 and Fig. 5).

**Table 1:** Outcomes of postoperative tissue-originated comprehensive genomic profiling

Genes	Mutation types	Mutation sites	Mutation abundance	
			1st operation	2nd operation
*PIK3CA*	missense mutation	exon21	3.73%	13.46%
*EGFR*	gene amplification	—	8.86 copies	30.33 copies
*NOTCH1*	missense mutation	exon6	1.27%	6.15%
*FAT4*	missense mutation	exon1	13.26%	16.89%
*STAG2*	frameshift mutation	exon26	17.66%	27.51%
*ATR*	missense mutation	exon13	42.68%	42.34%
*PRKDC*	nonsense mutation	exon77	43.48%	48.69%

*PIK3CA* phosphatidylinositol-4, 5-bisphosphate 3-kinase catalytic subunit alpha; *EGFR* epidermal growth factor receptor; *NOCTH1* notch homolog 1; *FAT4* FAT atypical cadherin 4; *STAG2* stromal antigen 2; *ATR* ataxia telangiectasia and Rad3 serine/threonine kinase; *PRKDC* protein kinase, DNA-activated, catalytic subunit

**Fig. 6:**
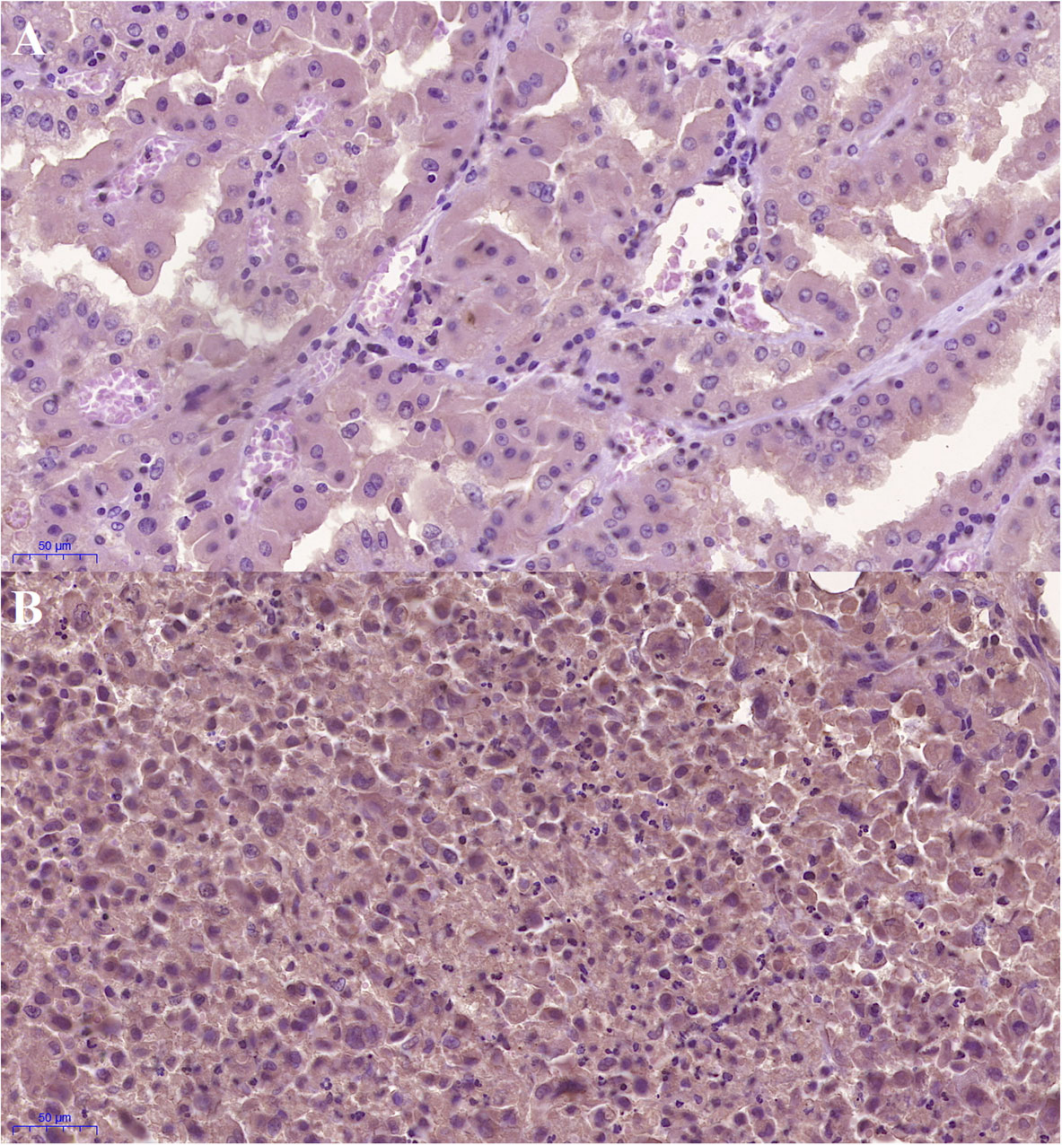
The immunohistochemistry for assessment of G-CSF expression in the renal tumor and in the specimen from the reoperation. Both of the staining outcomes revealed the positive expression of G-CSF. **a** Immunopositive for G-CSF from the specimen of 1st operation. **b** Immunopositive for G-CSF from the specimen of 2nd operation

Given some cases of G-CSF-producing RCC with leukemoid reactions have been reported previously [14, 15], the IHC for assessment of G-CSF expression in the renal tumor and in the specimen from the reoperation were performed on formalin-fixed paraffin-embedded tissue blocks. The blocks were sectioned into 4 μm and pretreated using PT Link (Dako, Glostrup, Denmark) with EnVisionTM FLEX Target Retrieval Solution High pH (Dako) containing Tris/EDTA buffer at pH 9.5. All washing was performed with EnVision FLEX Wash Buffer pH 7.75 (Dako). Staining was performed according to the manufacturer’s protocol (Dako). The samples were incubated with the primary mouse monoclonal anti-G-CSF antibody (Santa Cruz Biotechnology sc-53292, Dallas, TX, USA) at dilution 1:50 for 30 min. The secondary antibody incubation time was 15 min. All detection reagents were from the EnVision FLEX series by Dako (secondary antibodies, EnVision FLEX HRP, and EnVision FLEX Substrate Buffer). The slides were counterstained with hematoxylin (EnVision FLEX Hematoxylin) and dehydrated. Both of the staining outcomes revealed the positive expression of G-CSF (Fig. 6).

## Discussion

Renal tumor is rarely associated with ALR; only few papers reported several sporadic cases before [12–14]. ALR is probably caused by mechanical stimuli on bone marrow, resulting from bone metastases. They may also be caused by humoral stimuli resulting from neosynthesized blastic factors or factors released from the foci of tumor necrosis. Febrile sign is the clinical manifestation of ALR, possibly associated with paraneoplastic syndromes, resulting from either a release of endogenous pyrogens or tumor necrotic-inflammatory response [16], which decided the ineffectivity of antibiotic therapies.

Given the fact that RCC progression is usually accompanied with uncontrollable proliferation, distant metastasis, and recurrence, and the exact molecular mechanism of RCC is still unclear and needs to be further investigated, the genetic mutation analysis is becoming more and more important in the course of diagnosis and treatment for RCC. As for the outcome of postoperative tissue-originated comprehensive genomic profiling, the mutations of several vital oncogenes were indicated in this case. The *EGFR/PIK3CA* pathway has been reported as an important regulating role in the progression of RCC [17]. The expression of EGFR correlates with prognosis in patients with clear cell RCC [18], and suppression of the *EGFR* signaling pathway can retard the tumor progression [19]. Moreover, the *PIK3CA* pathway is highly activated in RCC progression [20]. *Ataxia telangiectasia and Rad3 Serine/Threonine Kinase (ATR)* responds to a broader spectrum of DNA damage and replication interference, including single-stranded DNA (ssDNA), double-stranded DNA (dsDNA) adjacent to ssDNA, adducts, cross-links, and inhibition of DNA polymerase [21]. This signaling pathway has a central role in detecting DNA damage, regulating DNA repair, and coordinating with other cellular processes. Its mutation can lead to genome instability and cell death [22, 23]. Besides, *protein kinase, DNA-activated, catalytic subunit (PRKDC)*, which is an important DNA-dependent protein kinase catalytic subunit, has been reported the over-expression in multiple human RCC tissues, and its inhibition via pharmacological inhibitors or siRNA/shRNA knockdown can significantly reduce the RCC cell proliferation in vitro and in vivo [24].

As for the relevance between ALR and our genetic findings, we found that the increased copy number of *EGFR* is due to polyploidy of chromosome 7. The *EGFR* copy number gain in tumor cells is associated with increased mutant allele transcription and gene activity. *EGFR* can activate the *RAS/RAF/MEK* pathway, which has a central role in the development and progression of cancer. A previous study reported that several growth factors including *EGFR* can induce G-CSF expression by a *RAS/RAF/MEK*-dependent mechanism, and the activation of the *RAS/RAF/MEK* signaling pathway triggered by *EGFR* can result in enhanced G-CSF expression and induce neutrophil recruitment significantly [25]. Another study reported the extreme leukocytosis and leukemoid reaction in a patient with lung sarcomatoid carcinoma. He underwent surgery for resection of the mass, and immunohistochemistry showed the overexpression of *EGFR*, but the authors did not further explore the potential relevance between *EGFR* and leukemoid reaction in this case [26]. In this case, we speculated that the possible mechanism is the proliferative mutation of *EGFR* induced the activation of RAS/RAF/MEK pathway and resulted in the upregulation of G-CSF, which caused ALR eventually. Besides, the *PI3K/AKT/mTOR* signaling pathway plays a central role in a wide spectrum of cellular activities, including cell proliferation, survival, and differentiation. Missense mutation of *PIK3CA* pathway components are involved in tumor development and occur in many cancer types. A previous study reported that inhibition of *PI3K* signaling decreases the production of proinflammatory cytokines including G-CSF [27]. Another study reported that G-CSF can activate the PI3K-Akt pathway in human neutrophils [28]. We speculated the possible mechanism driving the paraneoplastic leukemoid reaction in our case is through the interaction between *PIK3CA* and G-CSF, inducing a leukocytosis. In this case, the high intratumoral G-CSF expression and secretion, interacting with many oncogenes including *EGFR* and *PIK3CA*, might play a central role in the induction of ALR and could be viewed as an adverse prognosticator.

On the other hand, we speculate that the carbon dioxide pneumoperitoneum may be a potential risk factor for the tumor recurrence in this case. It has been a controversial issue whether the laparoscopic pneumoperitoneum enhances the tumor metastasis. Many reports addressed different conclusions based on their own outcomes [29–32], and there is no consensus until now. This rare case reminds us that high nuclear grading, which indicates high-grade malignancy, should be a warning sign to avoid choosing the laparoscopic or retrolaparoscopic surgery in RCC patients.

## Conclusions

ALR associated with overexpression of G-CSF may be a predictor of poor prognosis in patients with RCC, and comprehensive genomic profiling can help to provide potential valuable genetic etiological information and evidence for guiding the potential effective molecular-targeting therapy.

**Additional file 2:** Supplemental 2 Surgical video

## Supplementary information

**Additional file 1:** Supplemental 1

## Data Availability

All data generated or analyzed during this study are included in this manuscript.

## Abbreviations

ALR: Acute leukemoid reaction
RN: Radical nephrectomy
RCC: Renal cell carcinoma
WBC: White blood cell
CT: Computed tomography
RBC: Red blood cell
3D-DM: Three-dimensional digital model

## References

[1] Ljungberg B (2007). Prognostic markers in renal cell carcinoma. Curr Opin Urol..

[2] Siegel RL, Miller KD, Jemal A (2017). Cancer statistics, 2017. CA Cancer J Clin..

[3] Hollingsworth JM, Miller DC, Daignault S, Hollenbeck BK (2006). Rising incidence of small renal masses: a need to reassess treatment effect. J Natl Cancer Inst..

[4] Smith ZL (2016). Current status of minimally invasive surgery for renal cell carcinoma. Curr Urol Rep..

[5] Hoffman R, Furie B, McGlave P, Silberstein LE, Shattil SJ, Benz EJ, Heslop H (2009). Hematology: basic principles and practice. Volume 702. 5th edition.

[6] Riesenberg H, Müller F, Görner M (2012). Leukemoid reaction in a patient with adenocarcinoma of the lung: a case report. J Med Case Rep.

[7] Ma X, Li G, Cai Z, Sun W, Liu J, Zhang F (2012). Leukemoid reaction in malignant bone tumor patients- a retrospective, single-institution study. Eur Rev Med Pharmacol Sci..

[8] Boşoteanu C, Boşoteanu M, Aşchie M (2009). Differential diagnosis issues in a case of gastric carcinoma associated with leukemoid reaction. Rom J Morphol Embryol..

[9] Perez FA, Fligner CL, Yu EY (2009). Rapid clinical deterioration and leukemoid reaction after treatment of urothelial carcinoma of the bladder: possible effect of granulocyte colony-stimulating factor. J Clin Oncol..

[10] Saussez S, Heimann P, Vandevelde L, Bisschop P, Jortay A, Schandene L, Cogan E (1997). Undifferentiated carcinoma of the nasopharynx and leukemoid reaction: report of case with literature review. J Laryngol Otol..

[11] Qureshi KM, Raman AK, Tan D, Fakih MG (2006). Leukemoid reaction in pancreatic cancer: a case report and review of the literature. JOP..

[12] Huang W, Wang F, Li Y, Duan F, Yu Z (2006). Leukemoid reaction in sarcomatoid renal cell carcinoma: a two-case report. World J Surg Oncol. 2014; 12:100.Wang YC, Yang S, Tzen CY, Lin CC, Lin J. Renal cell carcinoma producing granulocyte colony-stimulating factor. J Formos Med Assoc..

[13] Sato T, Yamauchi N, Kobayashi D, Sato Y, Mochizuki C, Hori C, Watanabe N, Niitsu Y (1997). Granulocyte colony stimulating factor (G-CSF) producing renal cell carcinoma. Rinsho Ketsueki..

[14] Galia M, Albano D, Bruno A, Agrusa A, Romano G, Di Buono G, Agnello F, Salvaggio G, La Grutta L, Midiri M, Lagalla R (2017). Imaging features of solid renal masses. Br J Radiol..

[15] Sakka V, Tsiodras S, Giamarellos-Bourboulis EJ, Giamarellou H (2006). An update on the etiology and diagnostic evaluation of a leukemoid reaction. Eur J Intern Med..

[16] Liu F, Shangli Z, Hu Z (2018). CAV2 promotes the growth of renal cell carcinoma through the EGFR/PI3K/Akt pathway. Onco Targets Ther..

[17] Dorđević G, Matušan Ilijaš K, Hadžisejdić I, Maričić A, Grahovac B, Jonjić N (2012). EGFR protein overexpression correlates with chromosome 7 polysomy and poor prognostic parameters in clear cell renal cell carcinoma. J Biomed Sci..

[18] Liang L, Li L, Zeng J, Gao Y, Chen YL, Wang ZQ, Wang XY, Chang LS, He D (2012). Inhibitory effect of silibinin on EGFR signal-induced renal cell carcinoma progression via suppression of the EGFR/MMP-9 signaling pathway. Oncol Rep..

[19] Zhao Z, Liu H, Hou J, Li T, Du X, Zhao X, Xu W, Xu W, Chang J (2017). Tumor protein D52 (TPD52) inhibits growth and metastasis in renal cell carcinoma cells through the PI3K/Akt signaling pathway. Oncol Res..

[20] Zou L (2007). Single- and double-stranded DNA: building a trigger of ATR-mediated DNA damage response. Genes Dev..

[21] Cimprich KA, Cortez D (2008). ATR: an essential regulator of genome integrity. Nat Rev Mol Cell Biol..

[22] Abraham RT (2001). Cell cycle checkpoint signaling through the ATM and ATR kinases. Genes Dev..

[23] Zheng B, Mao JH, Li XQ, Qian L, Zhu H, Gu DH, Pan XD (2016). Over-expression of DNA-PKcs in renal cell carcinoma regulates mTORC2 activation, HIF-2α expression and cell proliferation. Sci Rep..

[24] Phan VT, Wu X, Cheng JH, Sheng RX, Chung AS, Zhuang G, Tran C, Song Q, Kowanetz M, Sambrone A, Tan M, Meng YG, Jackson EL, Peale FV, Junttila MR, Ferrara N (2013). Oncogenic RAS pathway activation promotes resistance to anti-VEGF therapy through G-CSF-induced neutrophil recruitment. Proc Natl Acad Sci U S A..

[25] Wang D, Zhang H, Yu F, Fang B (2016). Extreme leukocytosis and leukemoid reaction associated with the lung sarcomatoid carcinoma: an unusual case report. Int J Gen Med..

[26] Xie S, Chen M, Yan B, He X, Chen X, Li D (2014). Identification of a role for the PI3K/AKT/mTOR signaling pathway in innate immune cells. PLoS One..

[27] Kamata N, Kutsuna H, Hato F, Kato T, Oshitani N, Arakawa T, Kitagawa S (2004). Activation of human neutrophils by granulocyte colony-stimulating factor, granulocyte-macrophage colony-stimulating factor, and tumor necrosis factor α: role of phosphatidylinositol 3-kinase. Int J Hematol..

[28] Lin F, Pan L, Li L, Li D, Mo L (2014). Effects of a simulated CO2 pneumoperitoneum environment on the proliferation, apoptosis, and metastasis of cervical cancer cells in vitro. Med Sci Monit..

[29] Ishida H, Hashimoto D, Nakada H, Takeuchi I, Hoshino T, Murata N, Idezuki Y, Hosono M (2002). Increased insufflation pressure enhances the development of liver metastasis in a mouse laparoscopy model: possible mechanisms. Surg Endosc..

[30] Zheng L, Zhou D, Lu L, Liu Z, Fang L (2019). Effects of CO_2_ pneumoperitoneum on proliferation, apoptosis, and migration of gastrointestinal stromal tumor cells. Surg Endosc..

[31] Azuar AS, Matsuzaki S, Darcha C, Déchelotte PJ, Pouly JL, Mage G, Canis M (2009). Impact of surgical peritoneal environment on postoperative tumor growth and dissemination in a preimplanted tumor model. Surg Endosc..

[32] Zhang X, Guo X, Zhang A, Wang Y, Zhao J (2009). Influence of carbon dioxide pneumoperitoneum environment on adhesion and metastasis of a human ovarian cancer cell line. Surg Endosc..

